# Consumers’ willingness to pay premium under the influence of consumer community culture: From the perspective of the content creator

**DOI:** 10.3389/fpsyg.2022.1009724

**Published:** 2022-09-29

**Authors:** Jifan Ren, Jialiang Yang, Erhao Liu, Fangfang Huang

**Affiliations:** ^1^School of Economics and Management, Harbin Institute of Technology, Shenzhen, Guangdong, China; ^2^Shenzhen Gengya Technology Co., Ltd., Shenzhen, Guangdong, China

**Keywords:** consumer community, language and culture, value co-creation, WoPP, social comparison, consumer trust

## Abstract

With the rise of live streaming commerce, the relationship between consumers and content creators on the short-video platforms has become closer, forming a peculiar culture and language in each consumer community, which promotes the short-video platforms to become a natural breeding ground for forming consumer communities. While such communities give birth to its own language and culture from the interaction between content creators and consumers, this kind of co-creation can not only enhance the consumers’ trust to improve commodity premium space, but also strengthen the ties within the community and spread the information outside the communities, and consequently, expand community scale. Based on the view of the value co-creation from the language and culture among content creators and consumers in the communities, this study starts from the point of product type, employs consumers’ Willingness to pay premium (WoPP) as a proxy variable of brand advocacy in the co-creation of cultural and language values in consumer communities, and conducts three single-factor experiments between two groups. By analyzing the experimental results, this study identified the influence under the potential relationship mechanism, social comparison, and found another variable that can moderate the relationship, consumer trust, portrays the relationship between the product types of the live streaming commerce and the consumers’ WoPP, and explores the mediating effect of social comparison and the moderate effect of consumer trust effect. This paper also analyzes and discusses the WoPP caused by the co-creation of cultural and language values co-created by creators and consumer communities.

## Introduction

With the rapid rise of short-video platforms such as TikTok and Kuaishou, the overall social media environment has changed dramatically. Specifically, the short video as the main content of cultural creative virtual brand community has become an increasingly important source of cultural output. These short videos with different styles and themes meet the needs of users of different ages and social strata, conform to the consumption habits and requirements of modern society (Zhang, 2021), and provided the necessary conditions for the birth of consumer communities (Muniz and O'Guinn, 2001). Among three archetypes of community forms: business–consumer (BC), consumer–consumer (CC), and a combination of the two (BCCC), BCCC should be the ideal form (Storvang et al., 2020). Because of the rapid interaction may occur not only between content creators and consumers, but also among consumers, the consumer communities on content creation platforms should be categorized as the third form. Community is exactly one of the four dimensions that affects consumer behaviors (Mathras et al., 2016), and consumer communities on content creation platforms affect consumers in a special way. In such communities, the unique community culture and community language will be born in the interaction between content creators and consumers, and the unique language and culture co-created by such consumers and creators tend to enhance the trust of consumers in most of the time (Zhu et al., 2022), and promote consumption to feed creators back. The main consumption mode is live streaming commerce. It is captivating that the creation of short video content and the corresponding consumption mode of live streaming commerce need the continuous supply of high-quality content and marketing strategies, which makes the relationship between users and content creators closer. Thanks for the closer relationship, the consumer community becomes more active, and the frequency of community language and culture output is also increased. At the same time, the community will also expand in size due to the external communication of its language and culture (Weil, 1996). Values based on common language and culture are the foundation of community (Gardner, 1994). In this virtuous cycle, the value generated by the unique culture of the community created between content creators and consumers will not only be reflected in the improvement of the potential value of commodities, but also play a certain role in regulating consumers’ continued consumption in the creators’ community (Zhu et al., 2022). In other words, today’s short-video platforms are reshaping the traditional social media value co-creation process and giving value co-creation a whole new perspective and novel way of operating.

Nevertheless, research on the relationship between social media content creators and consumer value creation has not received extensive attention due to the relatively short rise of the market (Singaraju et al., 2016; Dolan et al., 2019). Furthermore, given that short-video platforms such as TikTok and Kuaishou have broken through traditional social media consumption patterns (e.g., expanding from influencer endorsements to live streaming commerce), emerging issues such as the interaction and connection between consumers and live streamers originally as content creators, how consumers understand the meaning and value of live streaming, and how content creators now as commodity agents balance economic benefits and customer relations have not yet been widely discussed in the academic community (Lu et al., 2021). Especially for value co-creation in social media, almost all traditional value co-creation studies seriously prefer to view value creation through non-interactive value formation methods when evaluating the concept of value creation (e.g., Bagozzi, 1975; Hunt, 1976). From this perspective, value is regarded as generated by separating the value systems of the company and the customer. And the value creation process is viewed as a firm-dependent phenomenon, as advocated by the Resource-based View (Barney, 1991). However, related research on marketing recognizes the importance of value creation from the perspective of interactive value formation (Prahalad and Ramaswamy, 2000; Vargo and Lusch, 2004). In contrast to embedding conceptualized value into products or services, “In this point of view, suppliers and customers jointly create services and shape products in cooperation. Moreover, it also means that value is not increased in separate and non-interactive production and consumption processes, but jointly created, realized, and evaluated in the social context of production and consumption processes.” (Echeverri and Skalen, 2011).

Based on such a perspective of value co-creation, systematically analysis of the social media content creators’ interaction with consumers, two-way influence, and community construction in commercial promotion activities is particularly important. From the perspective of TikTok content creators, this study will make an in-depth analysis of consumers’ coping strategies caused by differences in value co-creation elements when recommending different types of products. According to Yi and Gong’s (2013) theoretical measurement and analysis framework for customer value co-creation, this study takes consumers’ Willingness to pay premium (WoPP) for different live stream-recommended commodities (hedonic products and practical products)—whether they are willing to pay extra prices for recommended commodities—as a proxy variable for brand advocacy in consumer value co-creation behavior (Ranjan and Read, 2016) and explores the potential link through a series of online experimental studies.

## Literature review and research hypothesis

### Peculiar language and culture of each consumer community

Due to social demands and stress resistance, people will be continually distracted by social media (Koessmeier and Buttner, 2021). This phenomenon makes short-video platforms a natural breeding ground of consumer communities. According to the definition of brand community, “professional and non-geographically restricted community, based on the structured social relationship among the admirers of a brand” (Muniz and O'Guinn, 2001), the object of admiration of consumer community is not limited to a certain brand but can be all creators. The behaviors, systems, and norms generated in the community, and even extended to the knowledge, belief, art, law, custom, ability, and habit in the community, are collectively referred as the culture of the community (Tylor, 1871). At the same time, based on the common linguistic context, some communities’ unique languages emerged. On the one hand, such culture and language co-created by creators and consumers can have a positive impact on customer perceived value, which mediates the relationship between co-creation behavior and consumers’ continuous use intention (Zhu et al., 2022). In addition, the unique culture and language of the consumer community can stimulate the empathy of consumers in the community, which has a positive impact on consumers’ tolerance for services and their intention to purchase again (Wei et al., 2022). On the other hand, it can increase consumers’ trust in creators, thereby increasing their sense of trust in the products promoted and carried by creators (Zhao et al., 2021), and then feeding creators back. Rubio et al. (2016) once concluded on online consumption that because online shopping promotes competition, increases the comparison of homogeneous products, and reduces the cost for consumers to change decisions, consumers’ loyalty and trust in products are essential for online suppliers (Rubio et al., 2016). The main sources of consumer loyalty and trust include spokesperson, word of mouth, business leader, and business culture (Zhao et al., 2021). The language and culture of the consumer community is a great source of loyalty and trust for consumers.

On the other hand, the community will also expand due to the external communication of its language and culture (Weil, 1996). For example, recently many internet slangs from the short-video platforms are derived from some interesting, impressive punchline of content creators, and the content creators may use those slangs as their memory points or synonym. In this process, consumer communities will form their peculiar language and culture, and promote the expansion of communities according to such language and culture. Values based on a common language and culture are the foundation of community. Like what Gardner said, “no society can survive without a reasonable basis for shared values” (Gardner, 1994). Under this environment and background, the consumer community phenomenon is flourishing, and with the support of its unique language and culture co-created with the creators, it develops rapidly, laying the foundation for live streaming commerce.

If we observe the phenomenon of consumer community from the perspective of social practice theory, content creators, as an indispensable link in material, contribute to the participation and practice of platform users. In the traditional sense, consumer diffusion usually explores the flow of various phenomena (such as ideas, services, and information) by measuring the frequency of adoption, the mode of adoption, and/or the potential penetration level of adoption. Such studies of diffusion are usually conducted by tracking changes in sales in a particular market segment (Gatignon and Robertson, 1985). The penetration and diffusion of language and culture in a consumer community is exactly in line with this idea. Practice as a community-constructed concept can likewise diffuse or be diffused. Specifically, according to Akaka et al., 2022, practice theory includes the way social practices, working as links of understanding, action, and speech (Schatzki, 1996), are reproduced, stabilized, adapted, and embedded in a collection of fragmentary ideas (Reckwitz, 2002; Warde, 2005; Shove et al., 2012). It is important to explore how practices migrate (Maller and Strengers, 2013) and change (Shove, 2005) since such research helps to link up the previous consumer research on diffusion and practice. They provide an in-depth look and insight into the process of practice diffusion. For example, studies of the evolution of practice have explored questions such as how practices have changed over time, which further reveal the technical, social, and cultural factors that influence the integration and possession of routines, rituals, and cultures (Hand and Shove, 2004; Hand et al., 2005). Changes in practice can be studied within a particular social structure (e.g., family, group, and nation) or as they “cross” socio-cultural boundaries (e.g., transnational). Practice migration is a special way of practice dissemination and change (Maller and Strengers, 2013). In both cases, a practice can be adapted through shifts in broader socio-technical structures that include social, cultural, and material variety of products that shape everyday life (Hand et al., 2005). In this study, the unique culture and language created by the consumer community serves as the carrier of practice, which makes the practice spread within the community and migrate between communities, affirming the value created by the creator and the consumer community, and indirectly proving the premium space created by them.

### Experiential consumption and material consumption

In real life, products purchased by consumers can often be divided into different categories, among which experiential products and material products are typical classification methods (Gilovich and Gallo, 2020). Experiential products are those purchased primarily for the purpose of gaining life experience. On the contrary, material products refer to those products purchased for the main purpose of obtaining material wealth (Yang et al., 2020). Previous studies have found that compared with purchasing material products, purchasing experiential products can often bring consumers stronger happiness (Bastos and Brucks, 2017). Van Boven and Gilovich (2003) asked the subjects to recall the material products or experiential products purchased of more than $100 recently, and then asked them to indicate their perceived happiness from the purchased products. The final results showed that the subjects who recalled their purchase of experiential products perceived stronger happiness. Moreover, consumers tend to hold more negative evaluations of individuals who prefer to buy material products (vs. experientially purchased products) (Carter and Gilovich, 2010, 2012).

Based on the research, subsequent studies have explored the mechanisms behind experiential products (vs. material) making consumers happier. On the one hand, compared with experience, satisfaction and happiness brought by material consumers tend to fade away more easily. Although classical psychological studies have long found that individuals tend to show strong adaptive ability in both positive and negative experiences, their emotional reactions after corresponding events will gradually calm down (Kalokerinos et al., 2017). Nevertheless, relevant studies still show that compared with material purchase, the happiness and satisfaction brought by experiential products disappear more slowly (Nicolao et al., 2009). For example, in one study, people were asked to recall material or experiential purchases they had made for at least $50. When participants were asked how satisfied they felt at the time of purchase, there was no difference across conditions. Those who recalled material purchases enjoyed it as much as those who recalled experiential purchases. But when they are asked how satisfied they are with their purchases now, there is a clear difference: satisfaction with material purchases has decreased from then, but not with experiential purchases (in fact, satisfaction has increased) (Carter and Gilovich, 2010).

Another reason why experiential consumption is more likely to bring consumers stronger happiness is the consideration of interpersonal relationship. Compared with material consumption, experiential consumption can more closely connect consumers with others (Weingarten and Goodman, 2020), and then have a positive impact on the consumer community, which is conducive to the output of community language and culture. Previous research has shown that people who share similar experiences tend to have stronger bonds than people who buy the same products. It is obvious that consumers do not want others to buy the same products in terms of material purchases because consumers have strong unique demands for material products (Carter and Gilovich, 2010).

Finally, previous studies have compared experiential consumption and material consumption from the perspective of social comparison. Experience is less easy to compare than material products—it is more difficult to compare one by one to determine which one is superior, so it may be less affected by social comparison than material products (Boven, 2005). Similarly, Howell and Hill (2009) found in a study that experiential spending is related to the improvement of happiness partly because experiential consumption reduces social comparison.

### TikTok platform and consumer’s willingness to pay premium for material products

Although there is no research that directly explores and proves the influence of content creation platforms such as TikTok on consumers’ preference for different types of products, some studies provide a very important theoretical basis for the hypothesis of this research.

On the one hand, the use of media is closely related to materialism (Ferguson and Kasser, 2013). Some studies found that TV advertising was closely related to materialism (Shrum et al., 2011). Even after controlling for age, gender, and socioeconomic status, the relationship between television advertising and materialism remains significant (Buijzen and Valkenburg, 2003). Related research find (Shrum et al., 2005) that the world presented by television is quite different from the reality. For example, the level of wealth and achievement shown on television is greater. This may give viewers a distorted belief that wealth is quite common, which, in turn, may drive consumers to seek material wealth. On the other hand, the use of social media may increase consumers’ impulse buying behavior. Some studies have explored the psychological mechanisms behind this phenomenon. Social media intensity can lead to decreased self-control in individuals. Three potential sources of this failure include (1) emotional distress caused by goal conflict; (2) lack of self-awareness due to inability to monitor oneself; and (3) exhaustion of self-control ability due to the exertion of previous self-control (Baumeister, 2002). First, social media provides a deluge of information that can create conflicting standards about expected behavior. When people are not clear about which goals/standards/norms to pursue, they tend to adopt choices that make them feel good to reduce their pain. Therefore, the overuse of social media reduces consumers’ self-control. Second, browsing social networks enables people to follow the status and stories of other. As a result, it reduces their self-awareness. Declining self-awareness often leads consumers to have less control over themselves. Finally, self-control is similar to energy, which can be temporarily depleted and restored (Baumeister, 2002). Therefore, excessive participation in social media consumes self-control. On this basis, subsequent studies did find that social media use increased consumer impulse spending. Khan and Dhar (2006) found that consumers tend to display their luxury goods on social networking sites to boost their self-esteem. Similarly, when consumers want to achieve higher self-esteem, they will also show more indulgent behaviors (Wilcox et al., 2011).

Based on the existing studies above, this study believes that content creation platforms such as TikTok essentially meet the characteristics of traditional media and social platforms. Hence, consumers should have a significant preference on material products, and should willing to pay more premium for material product on those platforms compared with experiential products. More specifically, the recommendation of material products vs. experiential products in live streaming commerce on content creation platforms should significantly increase consumers’ WoPP. In conclusion, this study puts forward the first hypothesis:

*H1*: Compared with the experiential products, the material products recommended by the content creation platform have significantly increased consumers’ WoPP.

### The mediating effect of social comparison

According to Social Comparison theory (Festinger, 1954), individuals obtain evaluation information by comparing their own opinions and abilities with those of others. Social comparison tends to drive individuals to be better than others. Bogaerts and Pandelaere (2013) use social comparison to explain the internal mechanism of how individuals obtain group location information through social comparison. Lampi and Nordblom (2010) demonstrated that people are more concerned about their relative status if they are aware that their parents are constantly comparing them to others. The use of content creation platforms such as TikTok will increase consumers’ social comparison tendency. Nowadays, platforms such as TikTok have become a stage for beautiful people to show themselves, as well as a platform for individuals to “show off their wealth. Although the contents displayed are often embellished for the consideration of image management, these platforms are full of information indicating that others (and viewers) are doing better and living better (Chou and Edge, 2012). Such information can easily lead individuals to make upward social comparison. Researchers have demonstrated that the use of relevant platforms, especially passive use, significantly elicits upward social comparison (Vogel et al., 2014; Liu et al., 2017) and feelings of jealousy (Tandoc et al., 2015; Verduyn et al., 2015).

While classical psychological research considers social comparison to be informational reference, recent research has also found that social comparison profoundly influences individual product preference. For example, when the social comparison is high, consumers will prefer the products with design styles that can attract people’s attention (Rucker and Galinsky, 2009). In addition, previous studies have shown that there is an outstanding positive correlation between social comparison and materialism tendency. The stronger the social comparison tendency, the higher the materialism tendency of consumers. According to the social comparison theory, comparing with others who are better off will lead to unfavorable self-evaluation (Liu et al., 2017). In order to improve their impaired self-concept, individuals may try to have more material possessions to eliminate their insecurities. Researchers have found that individuals who make social comparison with media celebrities consider acquiring more property as their top priority (Chan, 2008).

From the perspective of social comparison, it is necessary to ensure that the products used have strong social comparison attributes. Experiential products emphasize pleasure and enjoyment; as a result, the comparability between products is poor. Material products emphasize functional and instrumental needs. Therefore, it is easy for individuals to find relatively better products, and it is easier to compare with others. Hence, the social comparison tendency increases the consumer’s preference for material products without affecting the consumer’s preference for experiential products. To sum up, this study puts forward another hypothesis:

*H2*: The relationship between the recommendation of material products in the content creation platforms and consumers’ WoPP is mediated by social comparison tendency.

### The moderating effect of consumer trust on willingness to pay premium

Previous marketing research literature constantly emphasizes the importance of mutual relations in business activities and business contacts in repeated and developed experiments, and advocates the need to establish a mutually beneficial and friendly relationship with customers (Gremler and Gwinner, 2020).

In fact, such a beneficial and mutually beneficial relationship can greatly enhance the sustainable transaction between the customer and the merchant, and ultimately achieve brand/company loyalty and review. Mutually beneficial relationships are largely based on consumer trust in the brand/company. Trust is a complex structure involving relationships among individuals, groups, and organizations (Fulmer and Dirks, 2018). In the process of conceptualizing trust, researchers from different fields have adopted different research perspectives. The differences between conceptualizations will cause confusion, misunderstanding, and communication barriers in the research of consumer trust (McKnight and Chervany, 2001). Nonetheless, sociological and organizational behavior research has focused the understanding of trust on a dynamic, reflexive process (Audrey Korsgaard et al., 2018; van der Werff et al., 2019). In this point of view, as demonstrated by the interactive relationship in the consumption process, trust is defined in this study as the confidence and positive expectation that the service provider (whether the company, the brand or the entrusted party represented by it) will fulfill the exchange agreement (Grayson and Johnson, 2015).

In the field of consumer behavior research, consumer trust has received extensive attention from scholars (Chaudhuri and Holbrook, 2001; Erdem and Swait, 2004). Based on different theoretical perspectives, different scholars have made in-depth discussions on the role of consumer trust in consumer decision-making, judgment, and behavioral tendency. Especially in recent years, the study of consumer trust along with the popularity of false information on the Internet and frequent brand crisis has gradually become an important research topic. For example, Kwon and Barone (2020) found through a series of experimental studies that exposure to fake news makes consumers with liberal tendencies more distrustful of information sources. At the same time, this distrust weakens their level of trust in the company providing the product/service, which lowers their evaluation of that product/service. Rajavi et al. (2019) investigated whether consumer trust in brands is influenced by the marketing mix activities (i.e., advertising, new product introductions, distribution, prices, and price promotions) implemented by brands using data from 46 product categories in 13 countries worldwide. They found that the intensity of advertising and new product launch had a strong positive impact on consumers’ brand trust, while the intensity of price and distribution also had a certain positive impact on consumers’ trust. However, the intensity of price promotion has a negative impact on consumer trust. In addition, the authors found that the impact of marketing mix activities on consumer trust is moderated by consumer personality traits, consumer dependence on a category brand, and national secular rationality and self-expressed cultural values. Trust not only affects consumers’ evaluation of businesses, but also affects the mutual trust between consumers. According to this inference, Engeler and Barasz (2021) found through myriad laboratory experiments and field experiments that brand collocation would affect consumers’ trust in another consumer’s recommendation. Specifically, consumers tend to lack trust in suggestions from other consumers using a single brand combination, and this distrust is determined by inferences about the way consumers choose products. With the iteration and change of the consumer scene, the consumer trust in e-commerce has gradually attracted the attention of the marketing community. Just as in traditional offline scenarios, higher consumer trust can also bring certain benefits to online sellers. The complexity and speed of online purchasing decisions make consumer trust an important determinant of the success of a business model (McKnight and Chervany, 2001; Paliszkiewicz and Koohang, 2013).

To sum up, this study believes that consumer trust has a moderating effect on the relationship between different types of products recommended by TikTok live streaming and consumers’ WoPP. Specifically, consumers’ WoPP for experiential products will decrease with the decline of consumer trust, but this moderating effect does not exist for material products.

*H3*: consumer trust produces a positive moderating effect on the relationship between product types and consumers’ WoPP, but this moderating effect does not exist in the material products.

**Figure 1:**
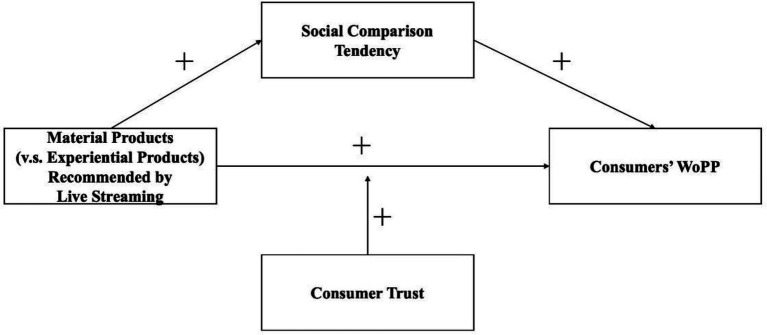
Theoretical model of research.

Based on the above Hypothesis, the research model of this paper is shown in Figure 1.

## Research design

### Experimental study one: Effect of material and experiential products on WoPP

The primary goal of Study 1 is to provide preliminary evidence for hypothesis 1 by experimental manipulation. The types of recommended products seen by the subjects were manipulated by experiment, and the WoPP was measured. A total of 211 Chinese adults, including 143 females, with an average age of 30.29 (SD =6.939), were recruited through the Chinese questionnaire collection platform (Questionnaire Star). The data sample service provided by this platform has been verified and supported by many studies, and its samples are representative to a certain extent, which can meet the sample characteristics and structure required by the research questions proposed in this study. Related studies on consumer behavior have also proved that the samples of the Questionnaire Star platform have certain advantages in terms of data reliability and validity (Guan et al., 2020).

**Figure 2:**
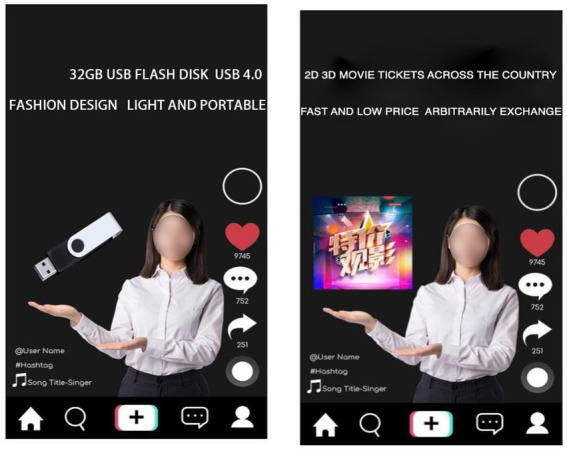
Study 1: Experimental materials.

Study 1 used a single-factor and two-level experiment process (TikTok content creators recommended product type: experiential vs. material), which experimentally manipulated the types of products the subjects saw and measured their WoPP for different types of products. In the study of operability, this study draws on the method of studying the behavior of the same type of consumer behavior, and uses the picture to present product information (To and Patrick, 2021; van der Lans et al., 2021). The subjects were randomly shown a screenshot of a TikTok content creator recommending a product. One typical material product and one typical experiential product were selected in this experiment. The subjects in the experiential product group saw an advertisement recommending movie tickets, while the subjects in the material product group saw a USB flash drive. The advertisement is shown in Figure 2. After viewing the corresponding product recommendations, the subjects were asked to indicate how much they agreed with the following statements: (1) Pay more for the product; (2) I can pay extra for this product; (3) It’s acceptable to pay more for this product; and (4) I am willing to pay more for this product (α = 0.939). Finally, after completing the purchase intention-related items, the subjects were required to complete the measurement of the manipulation test (“To what level do you think the product you just saw in the TikTok live streaming recommendation fits the following description?” 1, experiential product; 7, material products) and report their demographic information (income, gender, age, etc.).

**Figure 3:**
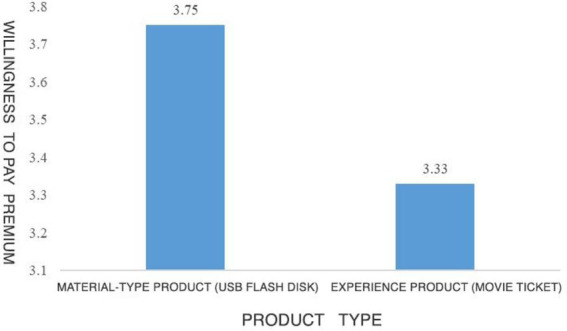
Result of study 1.

The subjects believed that the recommended products presented by the material product group had higher material attributes compared with the experiential products, while the experiential products had higher experience attributes. The difference was significant (M substance product = 1.59, SD = 0.62 vs. M experiential product = 5.88, SD = 0.58, *F*(1,201) = 3.668, *p* = 0.000). The manipulation of product types in this experiment is successful. The analysis of variance (ANOVA) result depicts that on the TikTok platform, in material products group, compared with experiential products, the subjects had higher WoPP (*M*_material products_ = 3.75, SD = 1.50 vs. *M*_experiential products_ = 3.33, SD = 1.58, *F*(1,201) = 2.376, *p* = 0.023). Therefore, hypothesis 1 is verified. Compared with experiential products, TikTok’s recommendation of material products increases consumers’ WoPP. The specific experimental results are shown in Figure 3. There were no significant differences in gender, age, and income between the two product types (*p* > 0.05).

In conclusion, through the experiment, Study 1 manipulates the types of recommended products subjects saw. The result portrayed that the WoPP of consumers on content creation platform is significant, and between consumers’ WOPP on USB flash drives and movie tickets, the WoPP of consumers exposed to the latter is significantly lower than the WoPP of consumers exposed to the former. Hypothesis 1 is verified: when recommending material products by content creators *via* content creation platforms, consumers’ WoPP is higher compared with recommending experiential products.

### Experimental study two: Mediating effect of social comparison

The above research verifies hypothesis 1: compared with recommendation of experiential products, content creators’ recommendation of material products on content creation platforms can increase consumers’ WoPP. Conversely, there are also some shortcomings: in addition to the material and experience differences, the two commodities content creators in Study 1 recommended themselves are not the same product (USB flash drive and movie tickets), so Study 1 cannot be ruled out the possibility that consumers are more willing to pay higher premium on USB flash drive rather than the movie tickets. In order to rule out such possibility, Study 2 will draw lessons from the previous experiential and material product research paradigm: packaging the same product into experiential products or material products (Goodman et al., 2019). In addition, Study 2 will verify the mediating effect of social comparison tendency. According to the hypothesis of this study, TikTok content creation platform improves the social comparison tendency of consumers, which, in turn, makes consumers more willing to pay higher premium for material products. A total of 225 Chinese adults, 134 of whom were female, with an average age of 32 (SD = 5.68), were recruited through questionnaire platform Credamo. Compared with Questionnaire Star platform, Credamo platform has the advantages of fast data collection, high questionnaire response rate, and better data quality. At the same time, the data collected by Credamo platform has been included in the data analysis by relevant studies, which proves that the platform has a good international recognition.

**Figure 4:**
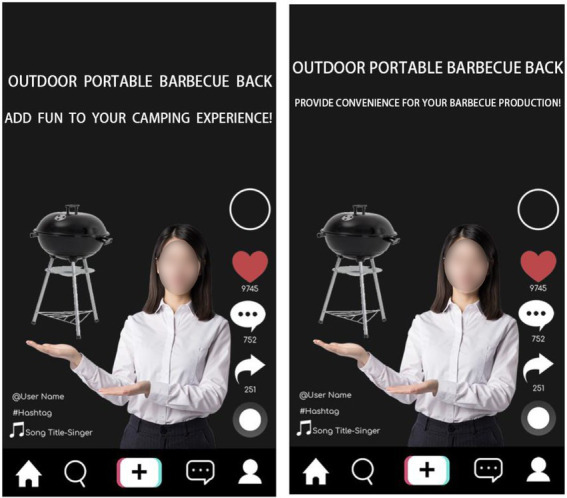
Study 2: Experimental materials.

Study 2 used a single-factor and two-level experiment process (TikTok content creators recommended product type: experiential vs. material), which experimentally manipulated the types of products the subjects saw and measured their WoPP for different types of products. The main process was basically consistent with Study 1. Major differences were that the subjects will see the different expressions of the same products, all subjects see the recommended products as a grill, but the material product group placed more emphasis on grill as a product in its own right, conversely the experiential group placed more emphasis on the nature of the grill to satisfy consumers’ experience together with family and friends (see Figure 4). The rest remained consistent. After viewing the corresponding TikTok-recommended products, the subjects need to indicate to what extent they agree with the following statements: (1) Pay more for the product; (2) I can pay extra for this product; (3) It is acceptable to pay more for this product; and (4) I am willing to pay more for this product (α = 0.895). The subjects’ social comparison tendency was then measured, and all of them were asked to say how much they agreed with the following statements at this moment in time: (1) I often compare my achievements in life to others; (2) If I want to know more about something, I try to know what other people think; (3) I always pay attention to: how am I doing things compared to other people; (4) I always compare what my loved ones (e.g., my boyfriend, girlfriend, family members, etc.) are doing with what other people are doing; (5) I always wonder what other people would do in a similar situation; (6) I’m not the kind of person who constantly compares myself to others; (7) If I want to know how well I am doing something, I compare what others are doing with what I am doing; (8) I often try to understand the opinions of others who are facing similar problems as I am; (9) In general, I like to discuss our opinions and experiences with others; (10) I never consider how my life compares to other people’s; and (11) I often compare my social performance (e.g., social skills, popularity) with others (Baldwin and Mussweiler, 2018; α = 0.903). Next, since all the products used in this experiment were barbecue grills, it was necessary to conduct manipulation tests on the types of products perceived by the subjects. All the subjects were required to answer their perception of the advertised products in the first task (1, totally material products; 7, totally experimental products). Finally, they report their demographic information (income, gender, age, and education).

**Figure 5:**
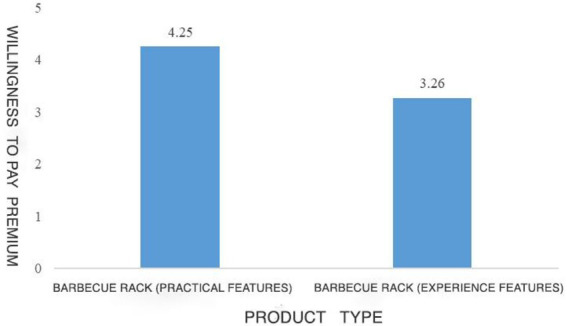
Result of study 2.

The material attributes perceived by the subjects in the material product group (*M* = 5.79, SD = 1.32) were significantly higher than those perceived by the subjects in the experiential product group (*M* = 1.64, SD = 1.68, *p* < 0.001), indicating that the manipulation of product types in this experiment was successful. The results of ANOVA convey that on TikTok platform, compared with experiential products, the subjects had higher WoPP on material product [*M*_material products_ = 4.25, SD = 1.78 vs. *M*_experiential products_ = 3.26, SD = 1.32, *F*(1,223) = 3.879, *p* = 0.014]. Therefore, hypothesis 1 is verified again. Compared with recommending experiential products on content creation platform, recommending material products is more capable to increase consumers’ WoPP. The experimental results are shown in Figure 5. This study first tested the influence of TikTok’s recommendation of material products (vs. recommendation of experiential products) on subjects’ social preference. The results of one-way ANOVA showed that compared with the subjects in the group of TikTok-recommended experiential products (*M* = 5.41, SD = 0.79), the subjects in the group of TikTok-recommended material products had a higher tendency of social comparison [*M* = 5.64, SD = 0.80, *F*(1,223) = 4.77, *p* = 0.03]. Then, this study conducted mediating effect analysis to test that TikTok-recommended material products lead to consumers’ higher WoPP by improving consumers’ social comparison tendency. For this purpose, the Model 4 mediation Model was applied in this study (Preacher et al., 2007), taking the WoPP as the dependent variable, the recommended product type as the independent variable, and the social comparison tendency as the mediating variable. The analysis results of the final mediation model show that social comparison can mediate the impact of product types recommended by TikTok content creators on consumers’ WoPP (95% CI [0.06, 0.28] excluding 0). Consequently, Hypothesis 2 is verified: content creation platforms’ recommendation of material products improves consumers’ social comparison tendency, and higher social comparison tendency, in turn, increases consumers’ WoPP for products.

To sum up, through the experiment, Study 2 revalidated hypothesis 1: compared with recommending experiential products on content creation platform, recommending material products is more capable to increase consumers’ WoPP. More indispensably, Study 2 verified the mediating effect of social comparison tendency: content creation platforms’ recommendation of material products significantly increases consumers’ social comparison tendency compared with content creation platforms’ recommendation of experiential products, and higher social comparison tendency conversely increases consumers’ WoPP.

### Experimental study three: The moderating effect of consumer trust

Study 3 focuses on the moderating effect of consumer trust to enrich the theoretical contribution of this study and identify the possible individual influencing factors of consumers in the process of purchasing goods on social media platforms. At the same time, this study will also exclude the influence of host gender on consumers’ WoPP for different product types. Previous studies have shown that the gender of spokespersons in advertisements may have a certain impact on consumers’ evaluation of products (Azar et al., 2018). As a result, different experimental designs will be carried out in this study to exclude the potential influence of host gender on the experimental results. Similar to Study 1, 200 Chinese adults were recruited through the Chinese questionnaire collection platform (Questionnaire Star), including 126 females, with an average age of 32.69 (SD = 7.358).

**Figure 6:**
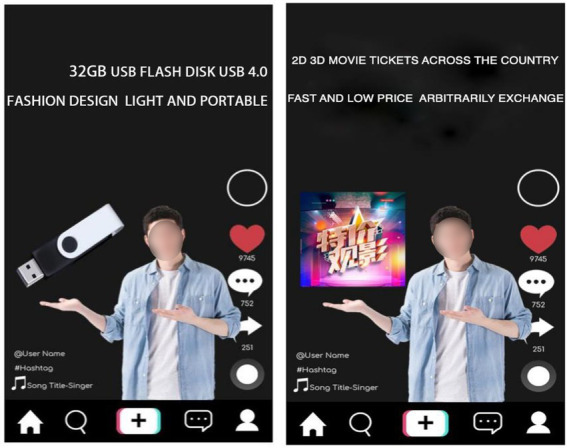
Study 3: Experimental materials.

Study 3 used a single-factor and two-level experiment process (TikTok content creators recommended product type: experiential vs. material), which experimentally manipulated the types of products the subjects saw and measured their WoPP for different types of products. In addition, this study will also measure the trust level of consumers (Yamagishi and Yamagishi, 1994). The main process of Study 3 was similar to that of Study 1 and Study 2. The subjects still saw two types of products: material products (USB flash drive) and experiential products (movie tickets). The advertisement is shown in Figure 6. The only difference between the experimental materials and the materials used in Study 1 is the host gender of the recommended product. In Study 1, the image of female recommender is used, while in Study 3, the image of male recommender is used to exclude the potential influence of gender on the product type effect. The specific product types and product descriptions were consistent with Study 1. After watching the pictures of TikTok’s product introduction, the subjects indicated their WoPP through the scale: to what extent did they agree with the following statements: (1) Pay more for the product; (2) I can pay extra for this product; (3) It is acceptable to pay more for this product; and (4) I am willing to pay more for this product (α = 0.912). After answering the WoPP, the subjects will receive a prompt informing that they are about to enter another study unrelated to the previous Tiktok questionnaire, in which the subjects will be asked some questions about their interpersonal relationship and communication. All the subjects need to answer to what extent they agree with the following views: (1) Most people are basically honest; (2) Most people are trustworthy; (3) Most people are basically kind; (4) Most people are trusting; (5) I always wonder what other people would do in a similar situation; (6) I trust people; and (7) When people are trusted by others, most of them will respond with kindness (Yamagishi and Yamagishi, 1994; α =0.904). Next, since the products used in this experiment are consistent with those in Study 1, for the sake of robustness, it is still necessary to conduct manipulation tests on the types of products perceived by the subjects. All the subjects need to answer their perception of the advertised products in the first task (1, completely material products; 7, totally experiential). Finally, they report their demographic information (income, gender, age, etc.).

**Figure 7:**
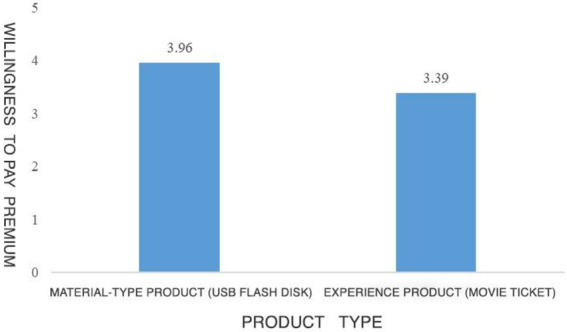
Result of study 3.

**Figure 8:**
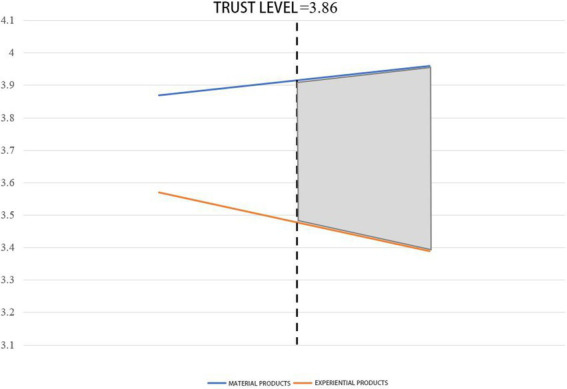
The moderation effect of trust.

Results showed that the material attributes perceived by the subjects in the material product group (*M* = 1.86, SD = 0.64) were significantly higher than those perceived by the subjects in the experiential product group (*M* = 6.02, SD = 0.54, *p* < 0.001). The manipulation of product types in this experiment is successful. The results of ANOVA showed that compared with TikTok-recommended experiential products, subjects had higher WoPP [*M*_material products_ = 3.96, SD = 1.37 vs. *M*_experiential products_ = 3.39, SD = 1.25, *F*(1,198) = 3.327, *p* = 0.012]. Therefore, hypothesis 1 is verified again. Compared with experiential products, TikTok recommends material products to make consumers more willing to pay premium. The experimental results are shown in Figure 7. At the same time, the main effect of the gender of TikTok hosts on the WoPP is not significant (*p* > 0.05), so the influence of gender on the results can be excluded. Then, the moderating effect of consumer trust as a moderating variable is analyzed. For this purpose, the Model 1 adjustment Model was applied in this study (Preacher et al., 2007), taking the WoPP as the dependent variable, the recommended product type as the independent variable, and consumer trust as the moderating variable. The analysis results of the final moderation model showed that consumer trust moderated the effect of product types recommended by TikTok content creators on consumers’ WoPP (*p* < 0.001). At the same time, the searchlight effect analysis shows that when the consumer trust level is 3.86, the difference in WoPP between material products and experiential products starts to emerge (see Figure 8). Therefore, hypothesis 3 is verified: consumers have a higher degree of trust in the material products recommended by content creators on content creation platforms, and consumers with a lower level of trust are less willing to pay product premium on experiential products.

In conclusion, through the experiment, Study 3 revalidated hypothesis 1 again: compared with recommending experiential products on content creation platforms, recommending material products is more capable to increase consumers’ WoPP. Different from the two studies above, Study 3 also verified the moderating effect of consumer trust: consumers with low levels of trust are more reluctant to pay product premium on experiential products. Furthermore, Study 3 also excluded a possible additional explanation—the gender of the host. The analysis results depict that gender has no significant impact on consumers’ WoPP.

## Discussion and conclusion

### Research conclusion

This study mainly focuses on the cultural value co-creation of creators and consumer communities in social media platforms and users’ WoPP, conducts an empirical test on the impact of product types on consumers’ WoPP, and analyzes the results according to relevant data. Based on the results of the discussions and the experimental studies, this study confirms the mechanism consumer communities influence consumers’ WoPP, portrays the relationship between the product types of the live streaming commerce and consumers’ WoPP, and explores the mediating effect of social comparison and the moderating effect of consumer trust effect.

Firstly, this study introduces the consumer community and its unique language and culture, demonstrates the value of the language and culture co-created by creators and consumers, and further discusses the penetration and expansion of practice based on this phenomenon as a carrier. Secondly, the research sorted out the relevant theoretical results and put forward the hypothesis of this research according to the relevant conclusions of the consumer behavior research, clarified the research content and design of this research, and adopted the behavioral experiment method to verify the research hypothesis. Specific research steps are as follows: In this study, consumers’ WoPP for different live-broadcast-recommended products (hedonic products and practical products) is taken as a proxy variable for brand advocacy in consumer value co-creation behavior (Ranjan and Read, 2016). Through a series of online experimental studies, we explore the potential connection between the two, identify the mechanism of action that affects the potential connection— social comparison— and find another variable that can moderate the relationship between the two— consumer trust.

Secondly, through the analysis results of three behavioral experiments, this study clearly shows the relationship between the types of products recommended by live streaming and consumers’ WoPP. When content creation platforms recommend material products, consumers are more willing to pay premium than when they recommend experiential products.

Finally, this study also explores the mediating effect of social comparison and the moderating effect of consumer trust, summarizes the results of hypothesis testing, and analyzes and discusses the mechanism of causality and potential boundary conditions. It is found that content creation platforms’ recommendation of material products significantly improves consumers’ social comparison tendency compared with content creation platforms’ recommendation of experiential products, and higher social comparison tendency, in turn, increases consumers’ WoPP. Consumers with low levels of trust are more reluctant to pay product premium on experiential products. Through the spread of consumer trust and social comparison in the consumer community, the author affirms the language and culture co-created by the creator and the consumer community, proves the potential value of its creation, and has a positive impact on consumers’ WoPP.

### Theoretical implication

This study mainly contributes to the existing literature from the following aspects: Firstly, this study confirms that the value of language and culture co-created by creators and consumer communities, and finds that different types of recommended products can significantly affect consumers’ value co-creation behavior—higher WoPP, which is helpful to better identify the relationship between product types and consumer value co-creation. Secondly, this study discovers the mediating effect of social comparison to further enriches the theoretical horizon of value co-creation, and provides a robust theoretical annotation for the potential links identified above from the perspective of causal inference. Finally, this study confirms the moderating effect of consumer trust which is also of practical significance for live streaming platforms. The moderating effect shows that building an advantageous trust mechanism through interaction with consumers, so as to better introduce the features and highlights of different products, can better improve the efficiency of live-streaming and thus increase profits.

Based on the three finding above, this study believes that the language and culture co-created by creators and consumer communities on content creation platforms, as the carrier of practice, can portray co-created value by consumers’ WoPP. Compared with experiential products, material products can lead to a higher WoPP. Furhter, the relationship between the recommendation of material products and consumers’ WoPP is mediated by social comparison tendency, and moderated by consumer trust.

### Practical application

Many people may think that more product types and choices should be added to the live broadcast room so that consumers can have more opportunities to contact more products, and consumers’ touch points should be increased through the launch and promotion of the live broadcast platform, to improve the sales efficiency and quality of the live broadcast room. However, this is not the case.

First, through the operation and maintenance of the consumer community, the creator creates the language and culture together with consumers, making it the “memory point” of the creator, which can promote the live broadcast of the creator more effectively and penetrate and spread in the consumer community, to contribute to WoPP. Second, just as many studies have found, material products and experiential products have distinct influences on consumers’ perception and evaluation, including consumers’ happiness (Boven, 2005), consumers’ specific evaluation of products (Gilovich and Gallo, 2020), and the willingness of consumers to write product reviews (Gallo et al., 2019). By precisely arranging the types of products recommended through live streaming commerce, consumers can pay more premium. Third, according to the conclusion of this study, it is not difficult to find that on content creation platform, since consumers can know all aspects of the society from various views, such a diversified online environment is likely to stimulate consumers’ social comparative psychology (Festinger, 1954). Also, the operation and maintenance of the consumer community, again, can contribute to a higher level of consumer trust.

After understanding such potential impacts, marketers and content creators engaged in live streaming commerce can better understand and recognize the nature of content creation platform, characters of consumer communities, and the properties of their own live stream, to make decisions that meet the needs of consumers and attract consumers and increase consumers’ WoPP.

### Research limitations and prospects

In this study, the three studies of product display are all recommend products on TikTok short-video platform. In future research, different platforms will be tried for product display, and a deeper inter-platform research will be conducted. Due to the short rise of the market, the quantitative measurement of concepts such as the co-creation value between creators and consumers and the degree of consumer trust is not exquisite enough. The measurement and quantification methods will be further explored in the future research to make a more refined, reasonable, and perfect quantitative comparison. Previous studies indicate that there may indeed be differences in user behaviors in different cultures. For example, considering the different relationship mobility between collectivism culture and individualism culture, there are differences in the content shared on relevant social media. Hence, accounting cross-cultural research in future research is salient and worth studying.

## Data availability statement

The raw data presented in this article are not currently available, because there are still ongoing researches based on them. The raw data will be available from the corresponding author upon reasonable request.

## Ethics statement

The studies involving human participants were reviewed and approved by the School of Economics Management, Harbin University of Technology, Shenzhen, China. Written informed consent for participation was not required for this study in accordance with the national legislation and the institutional requirements.

## Author contributions

JR, JY, and EL conceptualized the study and organized the data collection. JR and JY wrote the first draft of the manuscript. JY, EL and FH analyzed data and wrote the results section. FH revised important intellectual content and strengthened the research framework. All authors contributed to the article and approved the submitted version.

## Funding

This work was supported by the National Natural Science Foundation of China (grant number 71831005) and Shenzhen Humanities and Social Sciences Key Research Bases (grant number KP191001).

## Conflict of interest

FH was employed by Shenzhen Gengya Technology Co., Ltd.

The remaining authors declare that the research was conducted in the absence of any commercial or financial relationships that could be construed as a potential conflict of interest.

## Publisher’s note

All claims expressed in this article are solely those of the authors and do not necessarily represent those of their affiliated organizations, or those of the publisher, the editors and the reviewers. Any product that may be evaluated in this article, or claim that may be made by its manufacturer, is not guaranteed or endorsed by the publisher.
